# *In Situ* Hall Effect Monitoring of Vacuum Annealing of In_2_O_3_:H Thin Films

**DOI:** 10.3390/ma8020561

**Published:** 2015-02-06

**Authors:** Hans F. Wardenga, Mareike V. Frischbier, Monica Morales-Masis, Andreas Klein

**Affiliations:** 1 Surface Science Division, Department of Materials- and Earth Sciences, Technische Universität Darmstadt, Jovanka-Bontschits-Straße 2, Darmstadt 64287, Germany; E-Mails: hwardenga@surface.tu-darmstadt.de (H.F.W.); mfrischbier@surface.tu-darmstadt.de (M.V.F.); 2 Photovoltaics and Thin Film Electronics Laboratory, Institute of Microengineering, École Polytechnique Fédérale de Lausanne (EPFL), Rue de la Maladière 71b, CP 526, CH-2002 Neuchâtel 2, Switzerland; E-Mail: monica.moralesmasis@epfl.ch

**Keywords:** H-doped indium oxide, Hall effect, grain boundary passivation, hydrogen, X-ray photoelectron spectroscopy (XPS)

## Abstract

Hydrogen doped In_2_O_3_ thin films were prepared by room temperature sputter deposition with the addition of H_2_O to the sputter gas. By subsequent vacuum annealing, the films obtain high mobility up to 90 cm^2^/Vs. The films were analyzed *in situ* by X-ray photoelectron spectroscopy (XPS) and *ex situ* by X-ray diffraction (XRD), optical transmission and Hall effect measurements. Furthermore, we present results from *in situ* Hall effect measurements during vacuum annealing of In_2_O_3_:H films, revealing distinct dependence of carrier concentration and mobility with time at different annealing temperatures. We suggest hydrogen passivation of grain boundaries as the main reason for the high mobility obtained with In_2_O_3_:H films.

## 1. Introduction

Indium oxide is a wide band gap semiconductor commonly employed as transparent electrode material in electrochromic windows, flat panel displays, organic light-emitting diodes and solar cells [1–5]. It is mostly used as Sn-doped In_2_O_3_ (ITO). While high conductivities can be achieved with ITO, it has a high reflectivity in the infrared (IR) region of the light spectrum. The latter is a desired property for thermal insulation coatings for windows (low-E windows) [6,7] but it is a problem for devices that require near-IR (NIR) transparency, e.g., solar cells with an absorber material with an energy gap around 1 eV. Here, reflection of NIR light causes a reduction of the photocurrent of the solar cell. To achieve a transparent conductive oxide (TCO) film with a good NIR transparency, lower charge carrier densities (*n*) are required. To simultaneously still maintain high conductivities, the carrier mobility (*μ*) needs to be significantly higher. Koida *et al.* have shown that with hydrogen-doped In_2_O_3_ high carrier mobility (exceeding 100 cm^2^/Vs) can be achieved and reported a way to prepare such films by sputter deposition with the addition of H_2_O to the sputter gas and subsequent annealing in vacuum [8–10]. Employing such films as electrode for a-Si:H/c-Si heterojunction solar cells has been reported to improve solar cell efficiency [11,12]. Nevertheless, the origin of the high mobility obtained with In_2_O_3_:H films compared with undoped In_2_O_3_ films is still a matter of debate and will be further discussed in this work.

## 2. Experimental Section

Thin films of indium oxide were deposited on quartz glass substrates (CrysTec) by radio frequency magnetron sputtering from a ceramic In_2_O_3_ target. The target was purchased from MaTecK and had a purity level of 99.999%. The films were deposited using pure argon as sputtering gas under room temperature at a sputter power of 25 W and 0.5 Pa total pressure. To avoid unintentional heating of the substrate from the plasma, a target-to-substrate distance of 10 cm was used. Water vapor was added to the process gas during the deposition [8]. The water vapor partial pressure (*p*(H_2_O)) was introduced from a water reservoir, and the *p*(H_2_O) in the chamber was controlled by a leak valve. Due to *p*(H_2_O) being much smaller than the process gas pressure during sputtering, *p*(H_2_O) had to be adjusted before the introduction of the process gas into the chamber and could not be controlled during deposition. The mean value of the pressure before process gas introduction and the H_2_O pressure after the deposition (*i.e.*, when sputter gas was switched off) is used as *p*(H_2_O). Unfortunately, this results in a large error of *p*(H_2_O) of up to half an order of magnitude for some samples. Films with a thickness around 340 nm, measured via profilometry, were obtained with a deposition time of 75 min. Post deposition vacuum annealing was conducted at 200, 300 and 400 °C for 2 h in the deposition chamber with a base pressure of 10^−7^ mbar. A heating rate of 10 K/min was chosen.

The films were produced within the Darmstadt Integrated System for Materials Research (DAISY-MAT) [13]. The DAISY-MAT consists of several deposition chambers connected via an ultra-high vacuum distribution chamber to a photoelectron spectroscopy (PES) analysis chamber, enabling *in situ* XPS analysis of the deposited films without breaking vacuum. XPS measurements were conducted on a PHI 5700 spectrometer (Physical Electronics, Chanhassen, MN, USA). Monochromatic Al-K*α* radiation with an energy of 1486.6 eV was used for XPS measurements. A sputter-cleaned silver specimen was used to regularly calibrate the spectrometer.

Optical transmission measurements were conducted with a Lambda 900 spectrometer (PerkinElmer, Waltham, MA, USA). All transmission data shown in this work are in reference to the transmission of a blank quartz glass substrate. The *θ*–2*θ* XRD scans were measured on a SmartLab X-ray diffractometer (Rigaku, Tokyo, Japan). All XRD measurements were carried out in parallel beam geometry using Cu-K*α* radiation monochromated by a Ge-(220) × 2 channel cut monochromator on the primary side. A 5 mm Soller slit was employed on the secondary side to collimate the beam.

Hall effect and conductivity of the films were measured with the van der Pauw method at room temperature (RT) in air, as well as at temperatures up to 300 °C in vacuum. Therefore, a customized setup was used consisting of a homemade furnace situated inside the pole gap of an electromagnet. A quartz tube forming the furnace was joined via stainless steel glass-to-metal adapters to UHV DN16CF connectors. By connecting this furnace to a vacuum chamber, it is possible to evacuate the chamber and operate it at pressures down to 10^−8^ mbar. The furnace was heated by resistive heating from outside the glass tube to avoid sample contamination from the heating wire. Hall effect measurements were carried out with a magnetic field of 1 T. Further details of this setup are given in [14].

## 3. Results and Discussion

### 3.1. Hall Effect Measurements at Room Temperature

**Figure 1.**
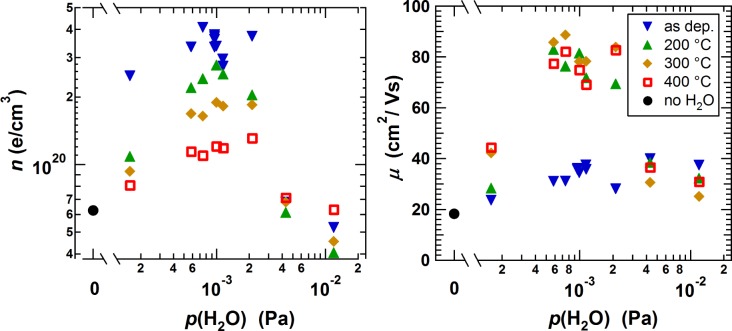
Hall effect measurements of In_2_O_3_:H films deposited with varying water vapor partial pressure and subsequently annealed at different temperatures. The circles correspond to non-annealed In_2_O_3_ film deposited at room temperature (RT) without additional H_2_O.

Room temperature Hall effect measurements of films deposited with *p*(H_2_O) between 1.6 × 10^−4^ Pa and 1.2 × 10^−2^ Pa were conducted. The values of *n* and *μ* are shown in Figure 1 for both as-deposited films and for films later annealed at 200, 300 and 400 °C for 2 h in vacuum. For comparison, *n* and *μ* of a non-annealed film deposited at RT without additional H_2_O is shown. The results are comparable to the results obtained by Koida *et al.* [8]. With the addition of H_2_O to the sputter gas, a significant increase of the carrier concentration for the as deposited films can be seen with a maximum of *n* = 4.1 × 10^20^ cm^−3^ at *p*(H_2_O) ≈ 10^−3^ Pa. For *p*(H_2_O) > 2 × 10^−3^ Pa, *n* decreases again. The former can be explained by an increasing donor doping with hydrogen as has been stated in literature [8,9] since H is expected to act as effective donor in In_2_O_3_ [15,16]. With increasing annealing temperatures *n* decreases, which is likely related to an increased degassing of H at higher temperatures [10]. Films deposited with *p*(H_2_O) around 10^−3^ Pa show a significant increase of *μ* from 30–40 cm^2^/Vs up to 90 cm^2^/Vs after annealing. This phenomenon will be discussed further below.

### 3.2. X-ray Diffraction

**Figure 2.**
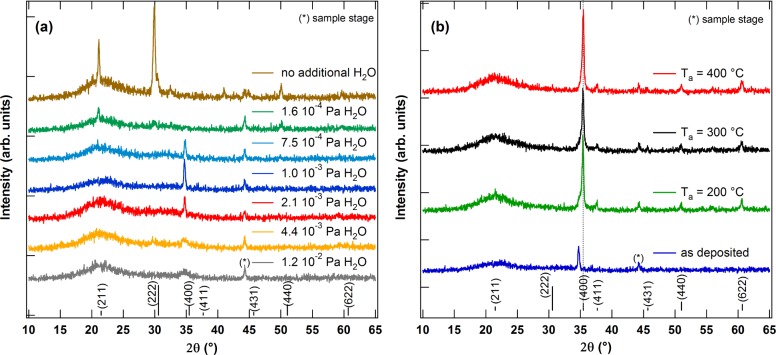
The *θ*–2*θ* diffractograms of as-deposited films with varying water vapor partial pressure (**a**) and films deposited with 1.0 × 10^−3^ Pa H_2_O after vacuum annealing for 2 h at different temperatures (**b**). Prominent reflections from In_2_O_3_ powder data (PDF 00-006-0416) are indicated for comparison.

In Figure 2a, *θ*–2*θ* scans of deposited films with varying *p*(H_2_O) are shown. Compared with the XRD pattern of the film deposited without additional H_2_O, which shows the characteristic reflections of the cubic bixbyite structure of In_2_O_3_, the reflection intensity decreases when H_2_O is added to the sputter gas. Only the (400) reflection with a low intensity can be seen for films deposited with *p*(H_2_O)≈ 10^−3^ Pa. A significant shift of the (400) reflection with *p*(H_2_O) cannot be seen. The suppression of crystallization by water addition to the sputter gas for In_2_O_3_ is well-known [8,9]. It is believed that the growth of crystallites is suppressed by adhesion of H atom, OH or H_2_O molecules at the growing surface [10,17]. After annealing in vacuum for 2 h at temperatures ≥ 200 °C (Figure 2b), the reflection intensity increases significantly, indicating crystallization during annealing as expected from literature [8]. In contrast to the results of Koida *et al.*, who reported films with a random crystallite orientation after crystallization [9], the films obtained in this work are strongly (100) oriented after annealing. Furthermore, a shift of the (400) reflection to higher 2*θ* values is observed. After annealing, the 2*θ* of the (400) reflections corresponds well with In_2_O_3_ powder data (PDF 00-006-0416). This indicates a relaxation of strain in the film during annealing and crystallization.

### 3.3. Optical Transmission Measurements

**Figure 3.**
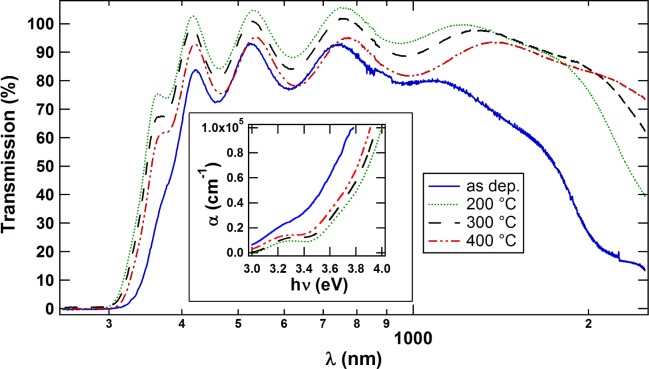
Transmission of In_2_O_3_:H films deposited with 1.0 × 10^−3^ Pa H_2_O before and after annealing for 2 h at different temperatures. Inset: corresponding absorption coefficient (*α*) at absorption edge.

Optical transmission measurements of the films deposited with *p*(H_2_O)= 10^−3^ Pa before and after annealing for 2 h at 200, 300 and 400 °C are shown in Figure 3. Compared with the as-deposited film, a higher NIR transmission is observed for the annealed films. This is considered to be due to a lower reflection caused by the lower carrier density in these films. In the visible region of the light all films show good transmission above 80%. The difference in the position of the absorption edges of the annealed films can also be attributed to the difference in carrier density. Note that *n* decreases with increasing annealing temperature, which results in a decreasing Burstein–Moss effect [1,6]. Thus, a decrease of the optical band gap can be seen for higher annealing temperatures. The absorption edge of the as-deposited film is at significantly lower energies. This is contrary to the Burstein–Moss shift for the annealed films, since the as-deposited film has the highest *n* and should therefore have a larger optical band gap than the annealed films. A possible explanation for this could lie in the as-deposited film being mostly amorphous. Due to the disorder in the amorphous phase, optical transitions that are usually parity forbidden in cubic In_2_O_3_ [18] could occur and lead to an optical band gap similar to the fundamental gap. A reduced band gap for a-In_2_O_3_ is also predicted by theory [19,20].

### 3.4. In Situ X-ray Photoelectron Spectroscopy

**Figure 4.**
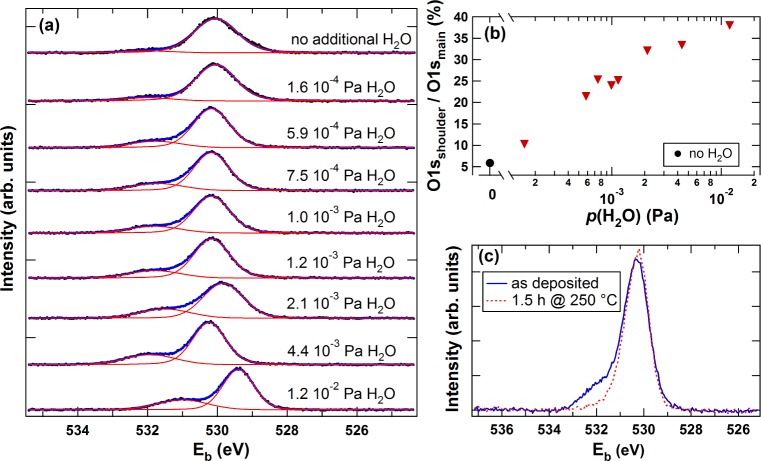
X-ray photoelectron spectra and fit of the O1s emission line of as-deposited In_2_O_3_ films with varying water vapor pressure (**a**), integrated intensity ratio of O1s shoulder-to-main emission line (**b**) and O1s emission lines of a film deposited with 1.0 × 10^−3^ Pa H_2_O before and after annealing in vacuum (**c**).

The results from the *in situ* XPS measurements of In_2_O_3_ films deposited with different *p*(H_2_O) are shown in Figure 4a. Shifts of the O1s emission lines with *p*(H_2_O) are parallel to shifts of the In3d core level and valence band maximum (data not included) and are therefore related to different Fermi level positions at the surface, excluding the sample deposited with *p*(H_2_O)= 4.4 × 10^−3^ Pa, which showed charging during the XPS measurement. These changes of Fermi level position are consistent with differences in carrier concentration as shown in Figure 1. The O1s emission lines of the films consist of a main emission line and a shoulder at approximately 1.7 eV higher binding energy, which can be attributed to In_2_O_3_ and In(OH)_3_, respectively [21]. Changes of the shape of the In3d emission lines cannot be observed for the films. According to Donley *et al.* the binding energy of the In3d level in In(OH)_3_ is at 444.8 eV [21], which is within 200 meV of the binding energy of the In3d level of our samples as well as for undoped In_2_O_3_ samples [5]. The presence of hydroxide therefore cannot be distinguished from In_2_O_3_ from the indium core level emission. For this reason, changes of hydroxide content with *p*(H_2_O) are estimated from the O1s emission lines in the following. The integrated intensities of the fits for the O1s shoulder and main emission are compared in Figure 4b. With increasing *p*(H_2_O), a significant increase of the shoulder intensity occurs. This indicates an increased hydroxide formation with increasing *p*(H_2_O). From the intensity ratio of shoulder-to-main emission, the hydroxide film thickness at the surface is estimated to be less than 1 nm. Following the route of formation, it is likely that hydroxide is also present in the interior of the film, but with a lower concentration than at the surface because the surface is exposed to H_2_O for some time after the deposition. After vacuum annealing, the shoulder emission disappeared (see Figure 4c), which can be explained by hydroxide decomposition during annealing. The decomposition of indium hydroxide is in good agreement with thermal desorption spectroscopy (TDS) measurements from Koida *et al.*, showing a pronounced H_2_O desorption peak at ∼135 °C and a broad desorption plateau at 220–380 °C [10]. The authors suggest that the peak of H_2_O desorption at ∼135 °C occurs with crystallization and originates from physically adsorbed and hydrogen bonded water molecules trapped at the surface of pores in the amorphous films. The broad desorption signal at higher temperatures is attributed to internal H_2_O related species in the film, which require diffusion to grain boundaries and/or the surface before desorption [10]. From our XPS results, we propose that the H_2_O desorption is likely related to the decomposition of In(OH)_3_ according to the chemical reaction 2 In(OH)_3_ → In_2_O_3_ +3 H_2_O. Desorption of H_2_O by the decomposition of indium hydroxide may induce void formation, which would agree with literature, where void formation has been observed in In_2_O_3_:H films after annealing [10].

### 3.5. In Situ Hall Effect Measurements during Vacuum Annealing

**Figure 5.**
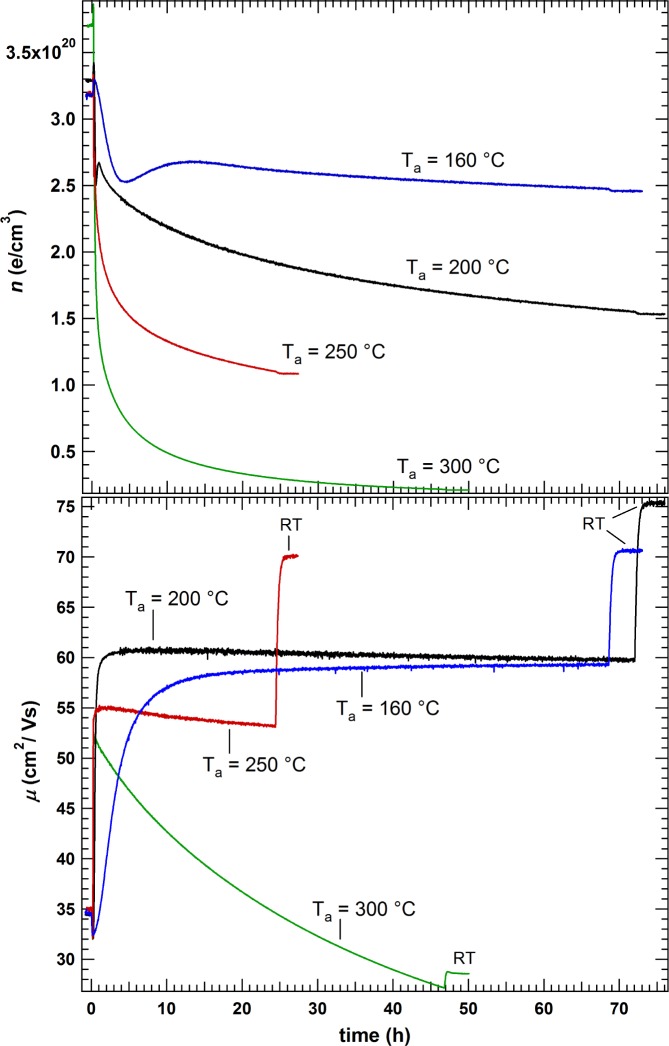
*In situ* Hall effect measurements during vacuum annealing at different temperatures of as-deposited films with *p*(H_2_O) = 1.0 × 10^−3^ Pa. All samples originate from the same deposition. The start of furnace heating is set to 0 h, with a rate of 20 K/min to reach the target temperature.

**Figure 6.**
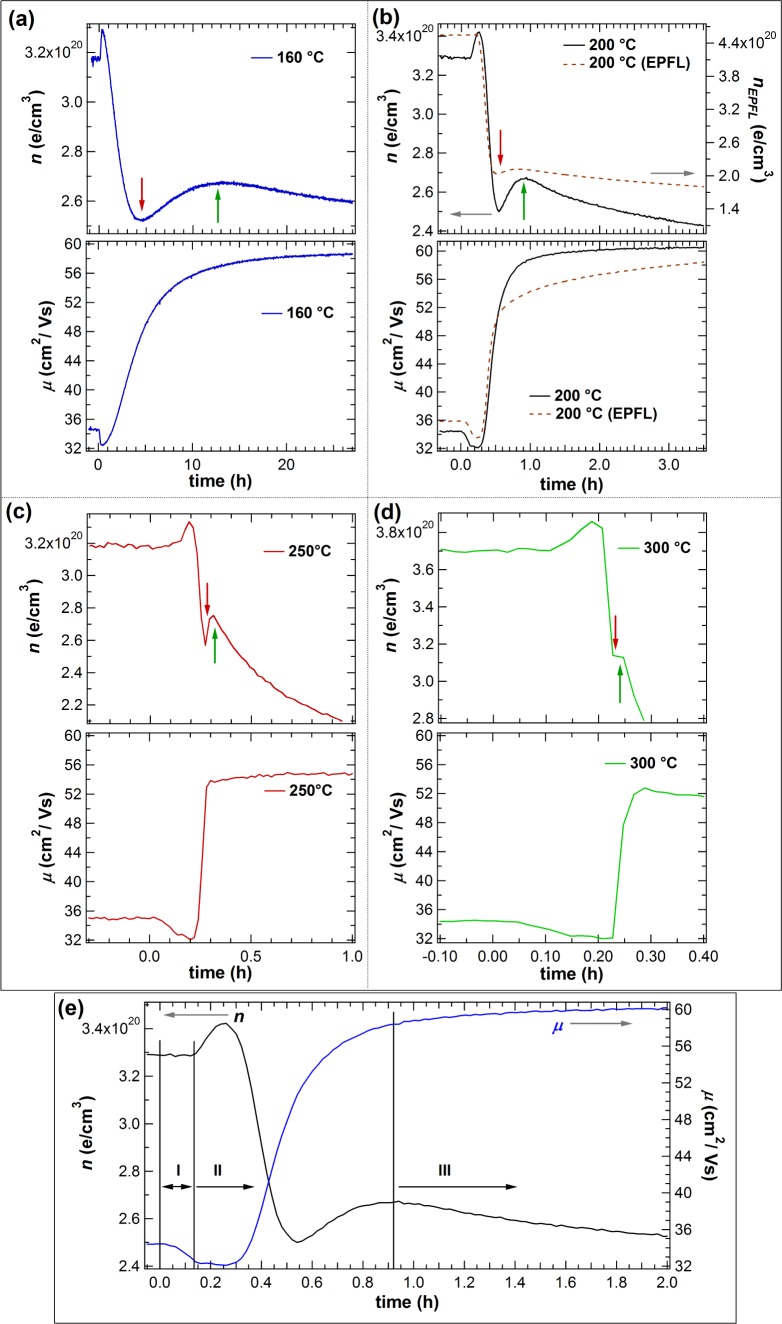
Zoom-in on the beginning of *in situ* Hall effect measurements during vacuum annealing at different temperatures of as-deposited films with *p*(H_2_O) = 1.0 × 10^−3^ Pa (**a**–**d**). Dependence of *n* and *μ* on time for the film annealed at 200 °C shown on a different timescale (**e**). The start of furnace heating is set to 0 h, with a rate of 20 K/min to reach the target temperature.

Non-annealed films from the same deposition with *p*(H_2_O) = 10^−3^ Pa were vacuum annealed at 160, 200, 250 and 300 °C. Hall effect measurements were conducted during annealing. The films were heated from room temperature to the annealing temperature (T_a_) at a rate of 20 K/min. After annealing for several hours the samples were cooled to room temperature. The measurements are shown in Figures 5 and 6. The start of the heating program is set to *t* = 0. Figure 6a–d shows the measurements at different annealing temperatures on different time scales in order to illustrate that a similar behavior is obtained independent of T_a_. The electron concentration first shows a small increase followed by sharp drop to a minimum before it rises again and goes through another maximum. The changes occur faster with increasing temperature until they are hardly distinguishable anymore for T_a_ = 300 °C. The carrier mobility shows an initial small decrease followed by a steep increase and a saturation for T_a_ < 250 °C. For T_a_ > 250 °C, the mobility decreases with longer annealing time. For comparison, the carrier concentration and mobility of a sample prepared under similar conditions at EPFL in Switzerland is included in Figure 6b. The slightly different behavior of *n* of this film could be related to the as-deposited film being completely amorphous and the smaller film thickness of 105 nm. It is noted that the film presents a preferential (111) orientation after annealing instead of the (100) orientation.

**Figure 7.**
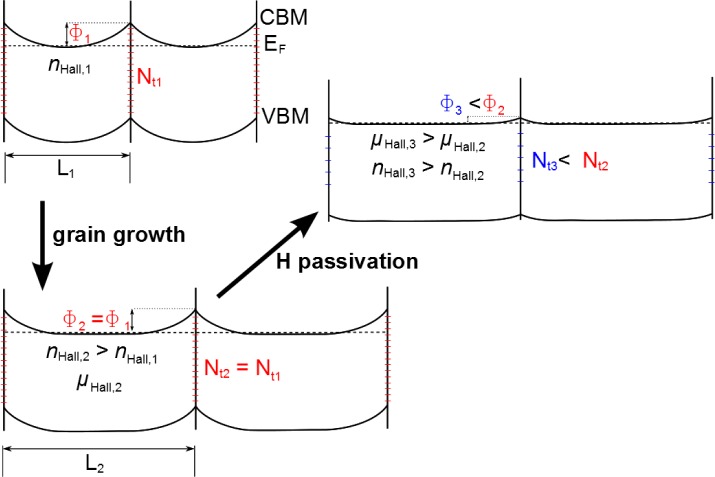
Schematic model of two neighboring grains with grain size L in order to elucidate the influence of grain growth on carrier concentration and the dependence of grain boundary states (N_t_) on transport barriers (*ϕ*) for electron transport.

In the following, a model is proposed to explain the observed changes of *n* and *μ* during vacuum annealing. For this purpose, the time evolution is divided into three steps (Figure 6e). *Step I* consists of the heating ramp of the furnace. Only a slight decrease in *μ* due to an increase of phonon scattering in the film can be seen, which is expected for degenerate semiconductors [22]. The electron concentration remains constant during this phase. In *step II* overlaying processes take place. There are at least two different processes with differing kinetics influencing *n*: one is increasing and the other is decreasing the carrier concentration. First, *n* increases while *μ* remains unchanged. It is suggested that this increase and the increase to the second local maximum of *n* is caused by the same effect, namely crystallization followed by grain growth. Considering a model of a polycrystalline material with depleted grain boundaries, it seems reasonable that for small grains, the depletion layer at the grain boundary could cause a reduction of the average carrier concentration in the grain. This effect would decrease with increasing grain size, resulting in an increase of effective carrier concentration (Figure 7). Crystallization and grain growth are considered to be superimposed by the decomposition of In(OH)_3_ that could release oxygen and hydrogen within the crystallizing film. A release of oxygen would decrease the donor defect concentration or increase the acceptor defect concentration, *i.e.*, the concentration of ${\text{V}}_{\text{O}}^{••}$ and $O_{i}^{\prime \prime }$ [23], respectively. We suggest that the release of O is responsible for the drop in carrier concentration. The hydrogen from the decomposition of In(OH)_3_ may saturate grain boundary states, thus leading to lower effective trap densities. This would decrease potential barriers at grain boundaries for electron transport (Figure 7). This mechanism is suggested as the main reason for the high mobility obtained in these films [9,24]. It is noted that for very small grain sizes a reduction of band bending and the depletion layer at grain boundaries could also be partly responsible for the increase of carrier concentration.

*Step III* includes a general decline of carrier concentration with time and increasing annealing temperature (see Figure 5). This is assigned to a degassing of H, hence a decreasing donor concentration in In_2_O_3_. For temperatures ≥ 250 °C, also the mobility decreases with annealing time. A reasonable explanation for this is the removal of H from the grain boundaries, resulting in non-passivated grain boundaries and higher transport barriers for electrons.

In literature, the observed high mobility of H-doped In_2_O_3_ films has been mostly ascribed to improved intra-grain properties due to the solid phase crystallization process, which leads to almost strain-free films and a lower concentration of strain-related defects [8,9], while a passivation of defects via H atoms, OH or H_2_O molecules has been mentioned only as a possibility [9]. Although the absence of strain, which is in good agreement with the XRD results shown in this work, and the removal of non-relaxed defects in the bulk might have an effect on the mobility, it appears unlikely as the main reason. The decrease of mobility at higher annealing temperatures with annealing time (Figure 5) is hardly consistent with such an explanation. The effect does not explain the decrease of *μ* for prolonged annealing times where defects and strain are rather removed than introduced. The model explaining the high mobility using H passivation of grain boundaries fits the obtained results and is in good agreement with literature, since grain boundary scattering is not expected to be important in high mobility H-doped In_2_O_3_ films [10]. In addition, the decrease of *μ* for T_a_ ≥ 250 °C due to removal of H from grain boundaries agrees with TDS results, which show an increase of H_2_ desorption around this temperature [10]. It is noted that film deposition at elevated temperatures with the addition of H_2_O or H_2_ to the sputter gas and post-deposition treatments in Ar/H_2_ mixtures did not increase the mobility sufficiently. Apparently, H is not incorporated in sufficient amounts or only in grains and not at grain boundaries. The hydroxide decomposition appears to be crucial for the H passivation of grain boundaries because it provides an internal source for hydrogen that is already present in the film during crystallization.

**Figure 8.**
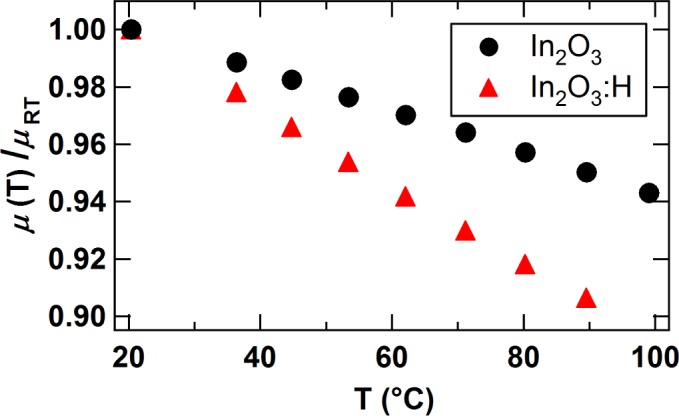
Carrier mobility at different temperatures normalized to mobility at room temperature (*μ*_RT_) for a post-annealed In_2_O_3_:H film (*μ*_RT_ = 84.8 cm^2^/Vs, *n*_RT_ = 1.06×10^20^ e/cm^3^) deposited at *p*(H_2_O)= 7.5×10^−^^4^ Pa and a polycrystalline undoped In_2_O_3_ film (*μ*_RT_ = 52.7 cm^2^/Vs, *n*_RT_ = 1.11 × 10^20^ e/cm^3^) deposited at 400 °C.

By comparing the mobility exceeding 100 cm^2^/Vs of single crystalline In_2_O_3_ films [25] and In_2_O_3_ single crystals [26] to the mobility of undoped polycrystalline In_2_O_3_ films (50–60 cm^2^/Vs), the lower mobility in polycrystalline films are likely related to grain boundary scattering [27,28]. The existence of transport barriers at grain boundaries in polycrystalline In_2_O_3_ films is supported by temperature-dependent Hall effect measurements. The results for an undoped and hydrogen doped film exhibiting nearly the same carrier concentrations are shown in Figure 8. With increasing temperature, a decline of the mobility is observed for both samples as expected due to enhanced phonon scattering [22,25]. However, the temperature dependence of the mobility is less pronounced for the undoped In_2_O_3_ film. Since both samples have comparable carrier concentrations, the influence of phonon scattering is expected to be identical for both samples [25]. An explanation for the different slopes of *μ*(T) could be an overlying effect that increases the mobility with temperature. Preissler *et al.* have shown that, for carrier concentrations comparable to those of the films in Figure 8, ionized impurity scattering is not significantly influenced by temperature changes [25]. A likely effect that would require a temperature activation could be electron transport across energy barriers at grain boundaries [29,30]. The occurrence of higher grain boundary barriers in undoped In_2_O_3_ compared with In_2_O_3_:H is likely the reason for the different slope of *μ*(T) and therefore supports the existence of grain boundary passivation via hydrogen.

## 4. Conclusions

**Figure 9.**
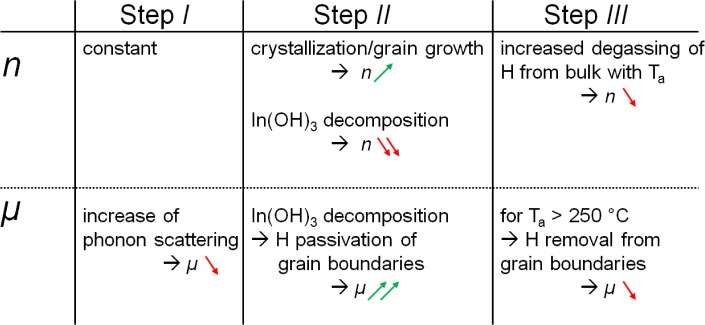
Summary of a model explanation for the changes in carrier concentration and mobility during the vacuum annealing of In_2_O_3_:H films.

The structural and electrical properties of H-doped In_2_O_3_ thin films and their optical transmission were investigated. From *in situ* XPS results, the occurrence of an In(OH)_3_ species that disappears during vacuum annealing has been found for the as-deposited films. Furthermore, results from *in situ* Hall effect measurements during vacuum annealing were shown, giving distinct dependence of *n* and *μ* on annealing time. A model explanation for the observed trends is proposed, which involves crystallization—more specifically grain growth—and hydroxide decomposition (Figure 9). As the main origin for the high mobility obtained in H-doped In_2_O_3_ films, hydrogen passivation of grain boundaries is suggested.
